# Targeting Impaired Nutrient Sensing via the Glycogen Synthase Kinase-3 Pathway With Therapeutic Compounds to Prevent or Treat Dementia: A Systematic Review

**DOI:** 10.3389/fragi.2022.898853

**Published:** 2022-07-18

**Authors:** Adrian Matysek, Sumudu Perera Kimmantudawage, Lei Feng, Andrea B. Maier

**Affiliations:** ^1^ Department of Human Genetics, University of Amsterdam, Amsterdam UMC, University Medical Centers, Amsterdam, Netherlands; ^2^ Department of Medicine and Aged Care, Royal Melbourne Hospital, Faculty of Medicine, Dentistry and Health Sciences, University of Melbourne, Melbourne, VIC, Australia; ^3^ Department of Psychological Medicine, Yong Loo Lin School of Medicine, National University of Singapore, Singapore, Singapore; ^4^ Healthy Longevity Translational Research Programme, Yong Loo Lin School of Medicine, National University of Singapore, Singapore, Singapore; ^5^ Centre for Healthy Longevity, National University Health System, Singapore, Singapore; ^6^ Department of Human Movement Sciences, Faculty of Behavioral and Movement Sciences, Amsterdam Movement Sciences, Vrije Universiteit, Amsterdam, Netherlands

**Keywords:** ageing, aged, Alzheiemer’s disease, dementia, cognition, glycogen syntase kinase 3, insulin resisitance

## Abstract

**Background:** Dementia is a global challenge with 10 million individuals being diagnosed every year. Currently, there are no established disease-modifying treatments for dementia. Impaired nutrient sensing has been implicated in the pathogenesis of dementia. Compounds that inhibit the glycogen synthase kinase-3 (GSK3) pathway have been investigated as a possible treatment to attenuate the progression of the disease, particularly the suppression of the hyper-phosphorylation process of the tau protein.

**Aims:** Systematically summarizing compounds which have been tested to inhibit the GSK3 pathway to treat cognitive impairment and dementia.

**Methods:** PubMed, Embase and Web of Science databases were searched from inception until 28 July 2021 for articles published in English. Interventional animal studies inhibiting the GSK3 pathway in Alzheimer’s disease (AD), Parkinson’s dementia, Lewy body dementia, vascular dementia, mild cognitive impairment (MCI) and normal cognitive ageing investigating the change in cognition as the outcome were included. The Systematic Review Centre for Laboratory animal Experimentation’s risk of bias tool for animal studies was applied.

**Results:** Out of 4,154 articles, 29 described compounds inhibiting the GSK3 pathway. All studies were based on animal models of MCI, AD or normal cognitive ageing. Thirteen out of 21 natural compounds and five out of nine synthetic compounds tested in MCI and dementia animal models showed an overall positive effect on cognition. No articles reported human studies. The risk of bias was largely unclear.

**Conclusion:** Novel therapeutics involved in the modulation of the GSK3 nutrient sensing pathway have the potential to improve cognitive function. Overall, there is a clear lack of translation from animal models to humans.

## 1 Introduction

The number of people aged 60 years and older is increasing globally, with a similar trend being seen in those affected by neurodegenerative diseases. In 2020, globally over 50 million people were living with dementia, of which 60–70% with Alzheimer’s disease (AD). Dementia has multiple causes and is characterized by impaired cognitive function. The neurodegenerative process of dementia begins a long time before the presentation of clinical symptoms (Katsuno et al., 2018). Factors contributing to the neurodegenerative process include metabolic disorders such as hyperglycaemia (Gale et al., 2018). As such it has been hypothesized that targeting ageing pathways may be a viable therapeutic option for treating dementia. One such pathway is the nutrient sensing pathway such as glycogen synthase kinase-3 (GSK3) which has been linked to the onset of dementias (Efeyan et al., 2015).

Impaired nutrient sensing is defined as dysregulation in processing nutrients for mammalian cells (de Lucia et al., 2020). Hyperglycemia such as in diabetes can lead to inflammation and apoptosis associated with high GSK3 activity. Hyperlipidemia and obesity can also increase GSK3 activity (Liu and Yao, 2016). The regulation of GSK3, one of the main molecules involved in insulin signalling (Jolivalt et al., 2008), plays an important role in this process. GSK3 is a protein kinase and present in many processes in organisms such as cell signalling and cellular transport (Souder and Anderson, 2019). It has two isoforms: alpha and beta. Both take part in glycogen metabolism and the phosphorylation process of over hundred substrates (Beurel et al., 2015), which are important for memory formation (Takashima, 2012). Impaired GSK3, when phosphorylated, induces hyper-phosphorylation of the tau protein, and subsequently lead to the formation of neurofibrillary tangles (NFTs) (Sayas and Ávila, 2021). Impaired GSK3 also contributes to the formation of amyloid beta (Aβ) plaques through the amyloid precursor protein (APP) cleavage pathway (Giese, 2009). Higher levels of GSK3 are associated with neuronal loss in Huntington’s disease and AD (L’Episcopo et al., 2016; Gale et al., 2018). Therefore, inhibition of GSK3 by therapeutic compounds could be a promising approach in managing dementia (Jope and Roh, 2006; Bhat et al., 2018).

This systematic review summarizes novel compounds inhibiting the GSK3 pathway in the context of preventing and treating cognitive impairment and dementia.

## 2 Methods

### 2.1 Selection of Articles

The protocol of this systematic review was registered at PROSPERO International prospective register of systematic reviews (Reg #: CRD42018091645). PubMed, Web of Science and Embase databases were searched until the 28 July 2021. Key search terms included Vascular Dementia (VD); AD; Lewy Body Dementia (LBD); Parkinson’s Disease (PD); cognitive ageing; autophagy; lysosome; ubiquitin; proteasome endopeptidase complex; molecular chaperone; unfolded protein response; insulin; mTOR; GSK3; protein kinase B (PKB)/Ak strain transforming (Akt); phosphoinositide 3-kinase (PI3K); 5′ AMP-activated protein kinase (AMPK); sirtuin; sirolimus; everolimus; temsirolimus; rapamycin; metformin; dipeptidyl peptidase 4 (DPP-4); glucagon-like peptide-1 (GLP-1); nicotinamide; nicotinamide adenine dinucleotide (NAD); spermidine; matinib; imatinib; nilotinib; dasatinib; bosutinib; ponatinib; bafetinib; lithium; heat-shock protein; caloric restriction; carbohydrate restricted diet; protein restricted diet; cognition. Additional relevant articles were identified by screening the references of included articles. After removing duplicates, remaining articles were screened for inclusion using the Covidence systematic review software (Veritas Health Innovation, Melbourne, Australia).

#### 2.1.1 Eligibility Criteria

Articles included in this review met the following inclusion criteria: 1) study population: animals or humans; normal cognitive ageing or dementia (AD, VD, LBD, PD). Populations likely to have a faster pace of cognitive ageing such as those with type 2 diabetes mellitus, insulin-resistance and obesity, were also included. For animals, normal ageing was defined as a strain not at a greater propensity to develop dementia and not manipulated to mimic dementia. Dementia models were defined as strains at a greater propensity to develop dementia compared to normal ageing strains. 2) Interventional study design with comparators, including randomised controlled trial, quasi-randomised controlled trials, and pre/post studies. 3) Intervention: compounds targeting cognition by inhibiting the GSK3 nutrient sensing pathway. 4) Outcome: cognitive function measured using standardized cognitive tests.

Articles were excluded if they met one of the following exclusion criteria: *in vitro* data only, conference abstract, review, editorial, or letter to the editor, or published in a language other than English.

### 2.2 Study Selection and Data Extraction

Two review authors (AM and SP) independently screened the titles and abstracts and subsequently the full text articles of potentially relevant studies against the inclusion and exclusion criteria. A third reviewer (ABM or LF) resolved any disagreements between the two review authors. All studies were divided into two groups: studies testing natural compounds (containing substances produced naturally by living organisms (Ouyang et al., 2014) and studies testing synthetic compounds.

The following variables were independently extracted for the included studies by two reviewers (AM and SP): author, year of publication, intervention, species/animal model, sample size (treatment group, control group), age, sex, duration of intervention, dose of therapeutic and type of model (dementia or normal ageing). For binary outcomes, the number of events, percentage of events or odds ratios with 95% confidence intervals were extracted. For continuous outcomes, the mean or median value, standard deviation, standard error, 95% confidence intervals or interquartile range, mean difference, and *p*-values were extracted.

### 2.3 Data Analysis

Interventions were considered preventative if the intervention was administered prior to the onset of dementia, with a delayed onset of dementia or reduced incidence rate after administration. Interventions were considered therapeutic if the intervention was administered after the dementia onset and subsequently slowed its progression or improved cognitive function.

An overall positive effect of the administered compound on cognitive performance was defined as positive finding on primary cognitive outcome, or >50% of the cognitive tests demonstrating a statistically significant improvement in the treatment group compared to the comparator group. A moderately-positive result was defined as ≥20% of the cognitive tests demonstrating a statistically significant improvement. A finding was considered negative where <20% of the cognitive outcomes were positive in the treatment group compared to the comparator group.

To investigate if the cognitive effect of compounds is dependent on study characteristics, studies were grouped based on study outcome (positive, moderately positive, negative), study population (MCI/dementia, normal ageing), the duration of intervention and the dosage of the compound.

### 2.4 Registered Clinical Trials in Humans

To identify ongoing and unpublished, completed human trials investigating GSK3 inhibitors, the ClinicalTrials.gov database (ClinicalTrials.gov, 2022) was screened for trials registered before 19 February 2022. Trials were included if GSK3 inhibition was mentioned, or compounds summarized in this review were utilized in combination with cognitive function, dementia, MCI, AD, VD, PD or LBD.

### 2.5 Risk of Bias

Two reviewers assessed the risk of bias (AM and SP). The Systematic Review Centre for Laboratory animal Experimentation’s risk of bias tool (SYRCLE) was used for animal studies (Hooijmans et al., 2014), which includes eight sources of bias: sequence generation, baseline characteristics, allocation concealment, random housing, blinding of personnel, random outcome assessment, incomplete outcome data, and selective outcome reporting. Results were denoted green for low risk, yellow for unclear risk, and red for high risk.

## 3 Results

### 3.1 Study Selection and Characteristics

**FIGURE 1:**
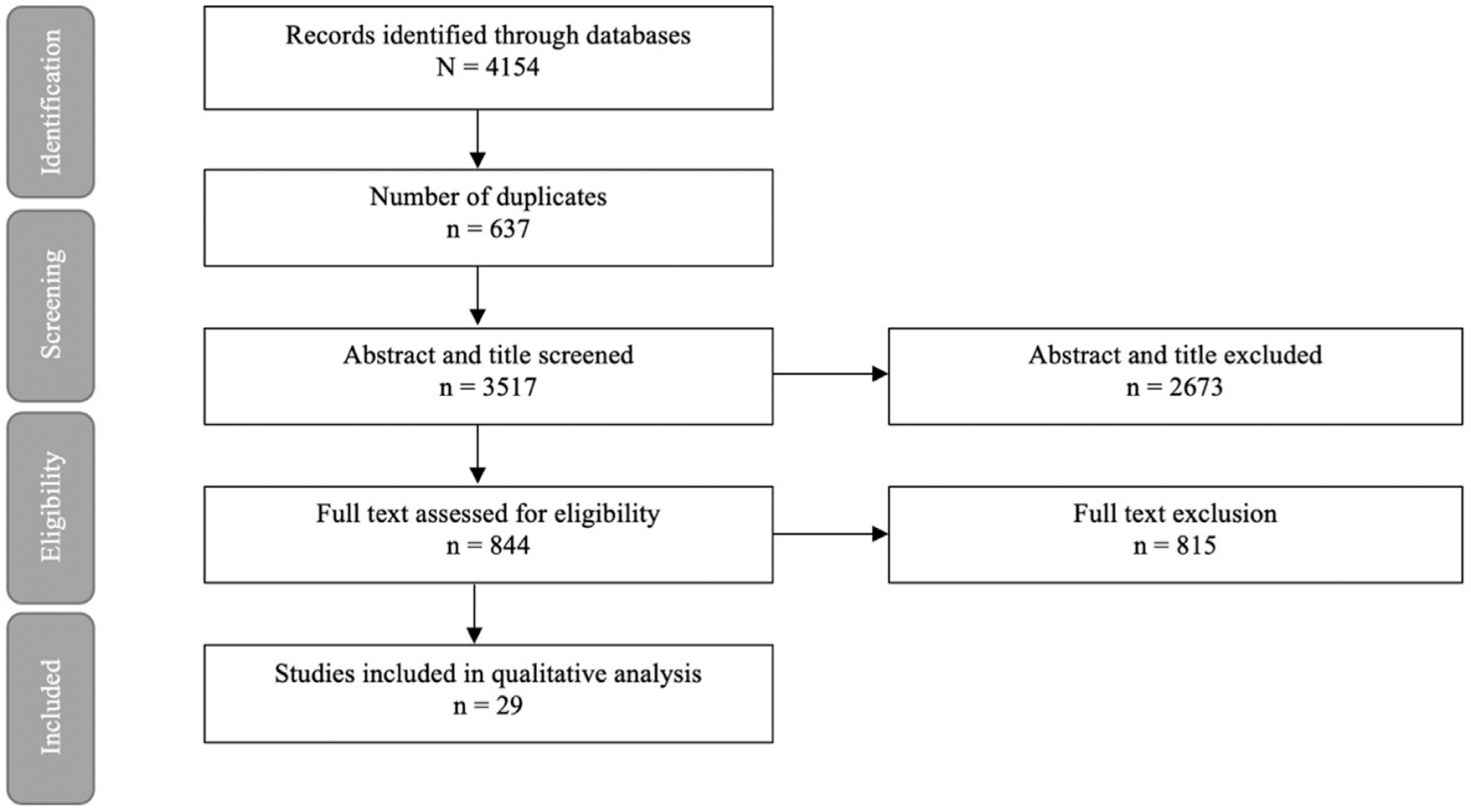
Article selection process.

**FIGURE 2:**
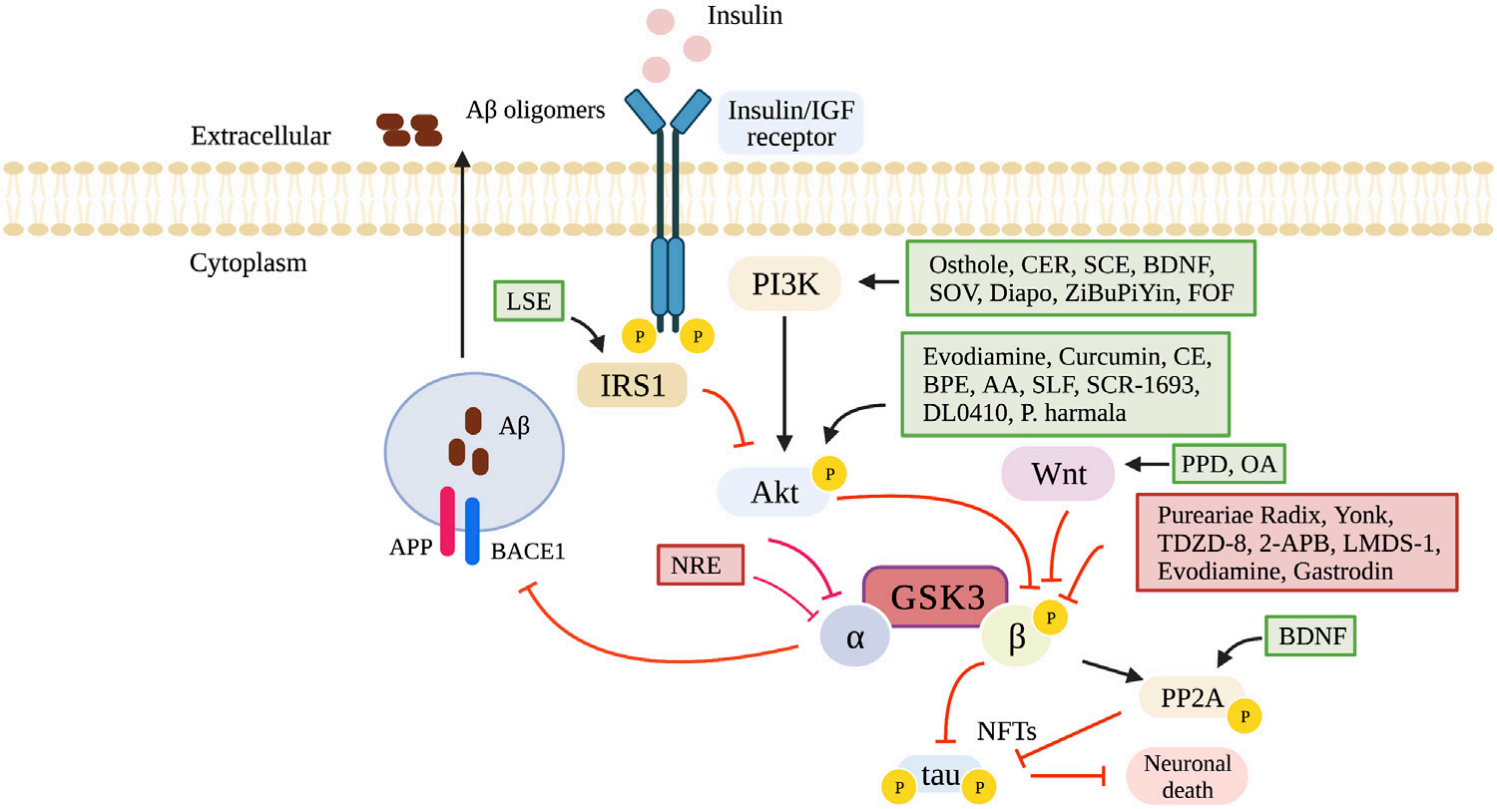
GSK3 pathway and its modulating compounds. 2-APB, 2-Aminoethoxydiphenyl borate; AA, Asiatic acid; Akt, Protein kinase-B (serine/threonine kinase); APP, amyloid precursor protein; BACE1, beta-site APP cleaving enzyme 1; BDNF, brain-derived neurotrophic factor; BPE, bee pollen extract; CE, cinnamon extract; CER, sea cucumber cerebrosides; Diapo, diapocynin; FOF, flavonoids of okra fruit; GSK3, glycogen synthase kinase-3; Insulin/IGF receptor, insulin-like growth factor 1 receptor; IRS1, insulin receptor substrate 1; NFTs, neurofibrillary tangles; NRE, N.incisum extract; OA, Oleanolic acid; P, phosphorylation; P. harmala, Peganum harmala; PPD, 20(S)-protopanaxadiol; PI3K, phosphoinositide 3-kinase; PP2A, protein phosphatase 2 (alpha isoform); SCE, Schisandra chinensis extract; SLF, seed of litchi chinensis fraction; SOV, sodium orthovanodate; Yonk, Yonkenafil. Created in BioRender.com.

The literature searches and selection process are illustrated in Figure 1. After exclusion of 4,154 duplicates, 3,517 articles were screened for title and abstracts of which 844 underwent full text screening. Thirty-two studies from 29 articles were included, investigating impaired nutrient sensing using compounds interfering with the GSK3 pathway on cognition in mice (19 studies), rats (12 studies) and zebrafish [one study (Koehler et al., 2019)] (Table 1). The Wistar rat was used most often to study dementia (8 out of 32 studies), whereas the C57BL/6 mice were used to study normal ageing [two studies (Jiang et al., 2020; Zhou et al., 2020)]. The sample size per treatment group varied between six (Xu et al., 2018; Lin et al., 2021) to 20 animals (Yang et al., 2013; Ibrahim et al., 2020). The majority of used animal dementia models were transgenic, overexpressing or producing mutant products of human genes such as amyloid precursor protein (APP) [Jiang et al., 2020; Zhou et al., 2020; Lin et al., 2021) and tau (Zhang et al., 2018)]. Dementia was also induced by administration of intracerebroventricular Aβ (Huang et al., 2019; Akhtar et al., 2020; Fan et al., 2020; Sun et al., 2020; Yan et al., 2021) or streptozotocin (Tang et al., 2018; Wang et al., 2018; Zhu et al., 2019; Akhtar et al., 2020). Cognitive tests used in the trials included Morris water maze, open field test, Barnes maze task, elevated plus maze, locomotor activity, Y-maze test, passive avoidance test, passive avoidance learning and novel object recognition test. Details of the tests are summarized in Supplementary Table 1. GSK3 inhibition was studied at different stages of the nutrient sensing pathway, which is illustrated in Figure 2.

**TABLE 1:** Characteristics of studies testing the effect of compounds interfering with the GSK3 pathway on cognition.

Author, year	Interventions	Species, model	Sample size (*n*)			Age, m	Sex, % F	Dura-tion, w	Dose	Cognitive/behavioural test(s)	Outcomes
			Rx	Ctrl							
Mild cognitive impairment or dementia											
Yang et al. (2013)	Yuzu extract	R, SD	20	20	NR		0	4	3% extract	MWM	
Madhavadas and Subramanian, (2017)	Aqueous cinnamon extracts	R, Wistar	8	8	2; 10		0	20	50 mg/kg	OFT; Barnes maze task	
Tang et al. (2018)	Lychee seed extract	R, SD	10–12	10–12	2–2,5		0	28d	0,7; 1,4; 2,8 g/kg	MWM	
Yan et al. (2017)	Schisandra chinensis extract	M, Kunming	10	10	NR		0	40d	300; 600; 1200 mg/kg	MWM; Y-maze; SPT; FST	
Zhang et al. (2018)	α-Lipoic acid	M, P301S	7	7	5		100	10	3; 10 mg/kg	MWM; OFT; NOR	
Koehler et al. (2019)	TDZD-8	Z, AB	12	12	12–15		50	10d	1 μM	Spatial alternation tasks	
Liao et al. (2019)	Bee Pollen Extract	M, CD-1	8–10	8–10	1,5		0	6d	30; 100; 300 mg/kg	MWM; Y-maze; PAT	
Li et al. (2019)	The sea cucumber cerebrosides	M, SD	8	8	NR		0	28d	40; 200 mg/kg	MWM	
SoukhakLari et al. (2018)	Curcumin	M, NMRI	NR	NR	1,64–1,97		0	10d	50; 100 mg/kg	Passive avoidance learning	
Wang et al. (2018)	Evodiamine	M, C57BL/6	15	15	4		NR	3	50; 100 mg/kg	MWM; NOR	
Yao et al. (2019)	Osthole	M, APP/PS1	8	8	6		100	8	20 mg/kg	MWM	
Xu et al. (2018)	BDNF	R, Wistar	6	6	1,6–1,8		0	NR	50 ng/ml	MWM	
Huang et al. (2019)	Puerariae radix	M, C57BL/6J	NR	NR	3		0	31d	340 mg/kg	MWM; Y-maze; OFT	
Jiang et al. (2020)	N.incisum extract	M, APP/PS1	9	6	6		0	NR	1 g/kg/d	MWM	
Ahmad Rather et al. (2019)	Asiatic acid	R, Wistar	12	12	NR		0	6	75 mg/kg	Elevated Plus Maze; Radial Arm Maze; OFT	
Sun et al. (2020)	Litchi chinensis seed fraction	R, SD	10	10	6		0	4	120; 240; 480 mg/kg	MWM	
Zhu et al. (2019)	Yonkenafil	R, Wistar	12	12	6–7		0	3	1; 3;10 mg/kg	MWM; Y-maze; OFT	
Akhtar et al. (2020)	Sodium orthovanadate	R, Wistar	8	8	NR		0	3	5; 10 mg/kg	MWM; OFT; NOR	
Bi et al. (2020)	SCR-1693	R, Wistar	7–12	7–12	NR		0	20d	1; 2;4 mg/kg	MWM	
Ibrahim et al. (2020)	Diapocynin	R, Wistar	20	20	NR		100	4	10 mg/kg	MWM; NOR	
Zhou et al. (2020)	DL0410	M, APP/PS1	NR	NR	9		NR	8	3; 10; 30 mg/kg	MWM	
Fan et al. (2020)	LMDS-1	M, C57BL/6J	12	12	2		0	4,7	5 mg/kg	OFT; Elevated plus maze; Y-maze	
Thapak et al. (2020)	2-APB	R, SD	8–10	8–10	NR		0	3	3; 10 mg/kg	Y-maze; MWM	
Saleh et al. (2021)	Peganum harmala	R, Wistar	13	13	NR		0	4	187,5 mg/kg	Y-maze; MWM	
Chou and Yang, (2021)	Evodiamine	M, ICR	5	5	2,5		0	4	50; 100 mg/kg	MWM; Passive avoidance learning	
Lin et al. (2021)	PPD	M, APP/PS1	6	6	6		0	4	10 mg/kg	MWM; Y-maze; OFT	
	Oleanolic acid	M, APP/PS1	6	6	6		0	4	10 mg/kg		
Ren et al. (2021)	ZiBuPiYin	M, C57BLKS/J-db/db	4	4	1,5		0	6	10 ml/kg	MWM	
Yan et al. (2021)	Flavonoids of okra fruit	M, Kunming	12	12	2,5		0	4	100; 300 mg/kg	MWM; Y-maze	
Wang et al. (2021)	Gastrodin	M, C57BL/6J	10	10	18		0	4	25; 50; 100 mg/kg	MWM	
Normal ageing											
Jiang et al. (2020)	N.incisum extract	M, C57BL/6; APP/PS1	9	5	AM; 6		0	NR	1 g/kg	MWM	
Zhou et al. (2020)	DL0410	M, C57/BL6J; APP/PS1	NR	NR	AM; 9		NR	8	3; 10; 30 mg/kg	MWM	

3xTg, triple transgenic; 2-APB, 2-Aminoethoxydiphenyl borate; AD, Alzheimer’s dementia; BDNF, Brain-derived neurotrophic factor; Ctrl, control; d, days; F, female; acid; MWM, Morris water maze; M, mouse; m, month; NR, not reported; NQ, not quantified; NOR, Novel Object Recognition; OFT, Open field test; PAT, perceptual ability test; PPD, 20(S)-protopanaxadiol; R, rats; Rx, treatment; SD, Sprague- Dawley; TDZD-8, 4-benzyl-2-methyl-1, 2, 4-thiadiazolidine-3, 5-dione; w, weeks; Z, zebrafish.

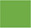
Primary cognitive outcomes are significantly positive or >50% of cognitive tests are positive; 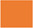
Overall ≥20% of cognitive outcomes tests are positive; 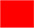
No significant change in any cognitive outcome or 20% of cognitive outcomes positive.

### 3.2 Natural Compounds

Twenty-two out of 32 studies tested naturally derived compounds, they include: compounds rich in flavonoids such as yuzu extract (Yang et al., 2013), bee pollen extract (Liao et al., 2019), N. incisum extract (Jiang et al., 2020) and flavonoids of okra fruit (Yan et al., 2021); compounds containing alkaloids, such as Puerariae radix (Huang et al., 2019), ZiBuPiYin (Ren et al., 2021), evodiamine (Wang et al., 2018; Chou and Yang, 2021) and Peganum harmala (Saleh et al., 2021); aquesous cinnamon extracts (Madhavadas and Subramanian, 2017), which contain type A linked procyanidin trimer; schisandra chinensis extract (Yan et al., 2017), which contains ligand diabenzo [a, c] cyclooctadiene; sea cucumber cerebrosides (Li et al., 2019), which is a class of neural glycospinolipids; osthole (Yao et al., 2019) which is a derivative of coumarin; curcumin (SoukhakLari et al., 2018), which is the main ingredient of turmeric; litchi chinensis seed fraction (Sun et al., 2020), which entails phenolic compounds and glycosides; Asiatic acid (Ahmad Rather et al., 2019), which is a pentacyclic triterpenoid; Yonkenafil (Zhu et al., 2019), which is a novel phosphodiesterase type 5 inhibitor and an analogue of sildenafil; 20(S)-protopanaxadiol, oleanolic acid (Lin et al., 2021), which is an active compound found in ginseng and a Chinese herb gastrodin (Wang et al., 2021).

Thirteen studies using natural compounds showed an overall positive effect (Yang et al., 2013; SoukhakLari et al., 2018; Tang et al., 2018; Ahmad Rather et al., 2019; Huang et al., 2019; Li et al., 2019; Yao et al., 2019; Jiang et al., 2020; Chou and Yang, 2021; Ren et al., 2021; Saleh et al., 2021; Wang et al., 2021; Yan et al., 2021) on cognition, seven studies a moderately positive (Madhavadas and Subramanian, 2017; Yan et al., 2017; Wang et al., 2018; Liao et al., 2019; Zhu et al., 2019; Sun et al., 2020; Lin et al., 2021) and one a negative effect (Jiang et al., 2020). The effect of natural compounds on cognition were tested in dementia animal models in rat (*n* = 7 out of 21 studies); mice (*n* = 14 out of 21 studies), and in normal ageing animal model in mice [*n* = 1 out of 1 study (Jiang et al., 2020)]. A dose-response relationship was examined in ten studies, of which seven studies (Yan et al., 2017, 2021; Wang et al., 2018; Liao et al., 2019; Zhu et al., 2019; Sun et al., 2020; Chou and Yang, 2021) showed that a higher dose led to better cognitive outcomes. The duration of administered natural compounds ranged from 6 days (Liao et al., 2019) to 20 weeks (Madhavadas and Subramanian, 2017) and did not affect the outcome.

The following compounds improved spatial memory: seed of litchi chinensis fraction, sea cucumber cerebrosides, evodiamine, osthole, Pureariae Radix, N.incisum extract, Asiatic acid, seed of litchi chinensis fraction, Yonkenafil, P. harmala (Tang et al., 2018; Wang et al., 2018; Ahmad Rather et al., 2019; Huang et al., 2019; Li et al., 2019; Yao et al., 2019; Zhu et al., 2019; Jiang et al., 2020; Sun et al., 2020; Saleh et al., 2021). Pureariae Radix showed improvement in recognition memory (Huang et al., 2019). Short-term memory was improved after administration of yuzu, schisandra chinensis extract, Pureariae Radix (Yang et al., 2013; Yan et al., 2017; Huang et al., 2019) and bee pollen extract showed improvement in long-term memory (Liao et al., 2019). Compound yuzu did not show any improvement in long-term memory (Yang et al., 2013). Motor activity did not improve after administration of schisandra chinensis extract and Yonkenafil, flavonoids of okra fruit (Yan et al., 2017, 2021; Zhu et al., 2019).

### 3.3 Synthetic Compounds

Ten out of 32 studies tested synthetic compounds including: SCR-1693 (Bi et al., 2020), an acetylcholinesterase inhibitor and calcium channel blocker; brain-derived neurotrophic factor (BDNF) (Xu et al., 2018); α-Lipoic acid (Zhang et al., 2018); diapocynin (Ibrahim et al., 2020), a NOX inhibitor, sodium orthovanadate (Akhtar et al., 2020), a tyrosine phosphatase inhibitor; DL0410 (Zhou et al., 2020) cholinesterase inhibitor; 2-APB (Thapak et al., 2020), which inhibit both inositol trisphosphate (IP3) receptors and transient receptor potential (TRP) channels; LMDS-1 (Fan et al., 2020), a potential TrkB receptor agonist and TDZD-8 (Koehler et al., 2019), a selective non-ATP competitive inhibitor of glycogen synthase kinase 3 beta (GSK3β).

Four studies using synthetic compounds showed an overall positive effect on cognition (Xu et al., 2018; Zhang et al., 2018; Akhtar et al., 2020; Ibrahim et al., 2020), five a moderately positive (Koehler et al., 2019; Bi et al., 2020; Fan et al., 2020; Thapak et al., 2020; Zhou et al., 2020) and one a negative effect (Zhou et al., 2020). The effect of synthetic compounds on cognition were studied in dementia animal models in rat [*n* = 5 out of 9 studies (Xu et al., 2018; Akhtar et al., 2020; Bi et al., 2020; Ibrahim et al., 2020; Thapak et al., 2020)], mice [*n* = 3 out of 9 studies (Zhang et al., 2018; Fan et al., 2020; Zhou et al., 2020)], zebrafish [*n* = 1 out of 9 studies (Koehler et al., 2019)] and in normal animal ageing model in mice [*n* = 1 out of 1 study (Zhou et al., 2020)].

Six studies (Zhang et al., 2018; Akhtar et al., 2020; Bi et al., 2020; Thapak et al., 2020; Zhou et al., 2020; Wang et al., 2021) investigated a dose-response relationship. For SCR-1693 (Bi et al., 2020), a higher dose gave better cognitive outcomes. The duration of administered compounds ranged from 10 days (Koehler et al., 2019) to 10 weeks (Zhang et al., 2018). No effect of the duration of the administrated compounds on cognition was found.

The following compounds improved spatial memory: α-Lipoic acid, BDNF, sodium orthovanadate, SCR-1693, diapocynin, DL0410, 2-APB (Xu et al., 2018; Zhang et al., 2018; Akhtar et al., 2020; Bi et al., 2020; Ibrahim et al., 2020; Thapak et al., 2020; Zhou et al., 2020). Sodium orthovanadate showed improvement in recognition memory (Akhtar et al., 2020). LMDS-1 improved short-term memory but not long-term memory (Fan et al., 2020). Motor activity did not improve after administration of the following compounds: α-Lipoic acid, sodium orthovanodate, diapocynin (Zhang et al., 2018; Akhtar et al., 2020; Ibrahim et al., 2020).

### 3.4 Human Registered Trials

No trials were found mentioning the inhibition of the GSK3 pathway and cognitive outcomes. Based on the compounds being described in this review, three human trials were found testing curcumin to improve cognition. One active, not recruiting phase 2 trial investigates curcumin (with aerobic yoga exercise) on levels of blood biomarkers and cognitive function in individuals with MCI or subjective cognitive impairment (NCT01811381). A completed phase 2 trials investigate the effect of curcumin on physical function and cognition in older adults (NCT03085680), and another phase 2 trial the effect of curcumin on mental capacity and amyloid-beta blood concentrations in AD patients (NCT01001637).

### 3.5 Risk of Bias Across Studies

Table 2 shows the SYRCLE risk of bias ratings for animal studies. All studies showed an unclear risk of bias on allocation concealment, random housing, blinding of personnel and random outcome assessment. A low risk was found across the sequence generation, baseline characteristics, incomplete outcome data and selective outcome reporting. Eleven studies showed unclear risk in the sequence generation criterion (Yang et al., 2013; Madhavadas and Subramanian, 2017; Tang et al., 2018; Wang et al., 2018; Ahmad Rather et al., 2019; Huang et al., 2019; Koehler et al., 2019; Liao et al., 2019; Akhtar et al., 2020; Thapak et al., 2020; Saleh et al., 2021). Unclear risk was mainly due to the lack of reported data.

**TABLE 2:** SYRCLE risk of bias for animal studies.

Author, year	Sequence generation	Baseline characteristics	Allocation concealment	Random housing	Blinding of personnel	Random outcome assessment	Incomplete outcome data	Selective outcome reporting
Yang et al. (2013)	Unclear	Low	Unclear	Unclear	Unclear	Unclear	Low	Low
Madhavadas and Subramanian, (2017)	Unclear	Low	Unclear	Unclear	Unclear	Unclear	Low	Low
Tang et al. (2018)	Unclear	Low	Unclear	Unclear	Unclear	Unclear	Low	Low
Yan et al. (2017)	Low	Low	Unclear	Unclear	Low	Unclear	Low	Low
Zhang et al. (2018)	Low	Low	Unclear	Unclear	Unclear	Unclear	Low	Low
Koehler et al. (2019)	Unclear	Low	Unclear	Unclear	Unclear	Unclear	Low	Low
Liao et al. (2019)	Unclear	Low	Unclear	Unclear	Unclear	Unclear	Low	Low
Li et al. (2019)	Low	Low	Unclear	Unclear	Unclear	Unclear	Low	Low
SoukhakLari et al. (2018)	Low	Low	Unclear	Unclear	Unclear	Unclear	Low	Low
Wang et al. (2018)	Low	Low	Unclear	Unclear	Low	Unclear	Low	Low
Yao et al. (2019)	Low	Low	Unclear	Unclear	Unclear	Unclear	Low	Low
Xu et al. (2018)	Low	Low	Unclear	Unclear	Unclear	Unclear	Low	Low
Huang et al. (2019)	Unclear	Low	Unclear	Unclear	Unclear	Unclear	Low	Low
Jiang et al. (2020)	Low	Low	Unclear	Unclear	Unclear	Unclear	Low	Low
Ahmad Rather et al. (2019)	Unclear	Low	Unclear	Unclear	Unclear	Unclear	Low	Low
Sun et al. (2020)	Low	Low	Unclear	Unclear	Unclear	Unclear	Low	Low
Zhu et al. (2019)	Low	Low	Unclear	Unclear	Unclear	Unclear	Low	Low
Akhtar et al. (2020)	Unclear	Low	Unclear	Unclear	Unclear	Unclear	Low	Low
Bi et al. (2020)	Low	Low	Unclear	Unclear	Unclear	Unclear	Low	Low
Ibrahim et al. (2020)	Low	Low	Unclear	Unclear	Unclear	Unclear	Low	Low
Zhou et al. (2020)	Low	Low	Unclear	Unclear	Unclear	Unclear	Low	Low
Fan et al. (2020)	Low	Low	Unclear	Unclear	Unclear	Unclear	Low	Low
Thapak et al. (2020)	Unclear	Low	Unclear	Unclear	Unclear	Unclear	Low	Low
Saleh et al. (2021)	Unclear	Low	Unclear	Unclear	Unclear	Unclear	Low	Low
Chou and Yang, (2021)	Low	Low	Unclear	Unclear	Unclear	Unclear	Low	Low
Lin et al. (2021)	Low	Low	Unclear	Unclear	Unclear	Unclear	Low	Low
Ren et al. (2021)	Low	Low	Unclear	Unclear	Unclear	Unclear	Low	Low
Yan et al. (2021)	Low	Low	Unclear	Unclear	Unclear	Unclear	Low	Low
Wang et al. (2021)	Unclear	Low	Unclear	Unclear	Unclear	Unclear	Low	Low

Sequence generation (allocation sequence adequately generated and applied); baseline characteristics (groups similar at baseline or adjusted for confounders in the analysis); allocation concealment (allocation adequately concealed); random housing (animals randomly housed during the experiment); blinding of personnel (caregivers and/investigators blinded from knowledge which intervention each animal received during the experiment); random outcome assessment (animals selected at random for outcome assessment); incomplete outcome data (incomplete outcome data adequately addressed); selective outcome reporting (reports of the study free of selective outcome reporting).

## 4 Discussion

Compounds inhibiting the GSK3 pathway may improve cognition in animals with cognitive impairment, but there is limited evidence for delaying cognitive decline or dementia onset in ageing animal models.

The GSK3 pathway can be inhibited at different stages of the nutrient sensing pathway. For the compounds osthole, sea cucumber cerebrosides, schisandra chinensis extract, brain-derived neurotrophic factor, sodium orthovanodate, diapocynin, ZiBuPiYin and flavonoids of okra fruit, GSK3β is inhibited by activation of the PI3K, and subsequent activation of Akt impaired GSK3 phosphorylation (Yan et al., 2017, 2021; Xu et al., 2018; Li et al., 2019; Yao et al., 2019; Akhtar et al., 2020; Ibrahim et al., 2020; Ren et al., 2021). The PI3K/Akt pathway is a key for regulating cell survival, growth and metabolism, which is important for cognitive function such as learning and memory (Shu et al., 2013). It plays an important role in apoptosis and autophagy in the nervous system, but also in reducing neuronal and nerve damage (Li et al., 2016). There is evidence that the insulin-PI3K/Akt pathway is reduced in AD, which makes compounds activating this pathway promising for the treatment of cognitive decline (Kong et al., 2013). Of the aforementioned compounds, osthole has been shown to inhibit the PI3K/Akt pathway via the activation of PTEN (Zhu et al., 2018). Brain-derived neurotrophic factor and diapocynin inhibits PI3K and AKT directly. Sodium orthovanadate inhibits PI3K and AKT downstream via the inhibition of PTP (Takada et al., 2004). The exact mechanism for sea cucumber cerebroside, and schisandra chinensis inhibition of this pathway remains uncertain.

The compounds evodiamine, curcumin, cinnamon extract, bee pollen extract, Asiatic acid, seed of litchi chinensis fraction, SCR-1693, DL0410 and P.harmala activate Akt, which leads to GSK3 phosphorylation impairment (Madhavadas and Subramanian, 2017; SoukhakLari et al., 2018; Tang et al., 2018; Wang et al., 2018; Ahmad Rather et al., 2019; Liao et al., 2019; Bi et al., 2020; Zhou et al., 2020; Chou and Yang, 2021; Saleh et al., 2021). Akt is important in protecting injured neurons (Huang et al., 2017) and promoting neurite outgrowth (Park et al., 2012). In early stages of neurofibrillary pathology as well as in hippocampal neurons in type 2 diabetes animal models, the levels of phosphorylated Akt are downregulated (Griffin et al., 2005; Xiang et al., 2015). Asiatic acid reduces oxidant mediated neuronal apoptosis, which leads to the inhibition of GSK3β (Ahmad Rather et al., 2019).

The compounds Pureariae Radix, Yonkenafil, TDZD-8, 2-APB, evodiamine, LMDS-1 and gastrodin directly modulate GSK3β (Wang et al., 2018; Huang et al., 2019; Koehler et al., 2019; Zhu et al., 2019; Fan et al., 2020; Thapak et al., 2020; Chou and Yang, 2021; Wang et al., 2021). Pureariae Radix prevents the decrease of inactive GSK3β Ser9 phosphorylation (Huang et al., 2019). Yonkenafil activates the phosphorylation of insulin receptor substrate 1 (IRS-1), leading to the phosphorylation of GSK3 (Zhu et al., 2019). TDZD-8 decreases the ratio of active/inactive GSK3β, and subsequently decreases pTau (Ser199) (Koehler et al., 2019). 20(S)-protopanaxadiol and oleanolic acid inhibit GSK3β via Wnt/GSK3β/β-catenin pathway activation (Lin et al., 2021).

The brain-derived neurotrophic factor activates protein phosphatase 2 (alpha isoform) (PP2A), which leads to a decrease in p-Tau. The result is a dephosphorylation of active GSK3β and an enhancement of inactive GSK3β Ser9 (Xu et al., 2018). Compound N. incisum extract modulates extracellular Aß production caused by unphosphorylated GSK3β. Specifically, it suppresses the expression of the cleaving enzyme beta-site APP cleaving enzyme 1 (BACE1), preventing the cleavage of GSK3 substrate APP. The result is a decrease in Aß production and subsequently the decrease of p-Tau and neuronal death (Jiang et al., 2020). Compound lychee seed extract reduces the deposit of Aß associated with insulin dysfunction and resistance (Tang et al., 2018). IRS-1 is activated by catechin, procyanidin A1 and procyanidin A2 derived from lychee seed extract. The result is the inhibition in the activity of GSK3β in the PI3K/Akt/GSK3 pathway (Xiong et al., 2020).

The GSK3 pathway is one of the multi-aetiological factors implicated in insulin resistance (Leng et al., 2010), which might be of importance as more than 80% of patients with AD have either type 2 diabetes mellitus or an impaired fasting glucose (Barbagallo, 2014). Disturbed glucose metabolism has also been shown to increase the risk of developing dementia by 50% (Biessels et al., 2014).

Inflammation is one of the main features observed in both type 2 diabetes mellitus (Tsalamandris et al., 2019) and AD as well as other neurodegenerative diseases (Guzman-Martinez et al., 2019). Yang et al. (2013), Ahmad Rather et al. (2019), Huang et al. (2019), Yao et al. (2019), Zhu et al. (2019), Ibrahim et al. (2020), Jiang et al. (2020), Sun et al. (2020), Zhou et al. (2020), Chou and Yang (2021), Yan et al. (2021) of the investigated compounds have anti-inflammatory properties in additional to their GSK3 inhibitory properties. Low grade systematic chronic inflammation is a hallmark of human ageing and an impaired immune response. Therewith, anti-inflammatory properties could be another important pathway contributing to the cognitive benefits of such compounds (Tangestani Fard and Stough, 2019).

Four studies investigated N. incisum extract, Yonkenafil and DL0410 showed that these compounds decrease necrosis factor alpha (TNF alpha) or interleukin 6 (IL-6). It is noted that levels of these two cytokines are elevated in AD and type 2 diabetes mellitus (55). TNF alpha activates the c-Jun kinase (JNK), which leads to the inhibition of serine phosphorylation of IRS and blockade of insulin signalling (Gao and Ye, 2012). Interestingly, high levels of insulin lead to increasing levels of TNF alpha and IL-6. Hyperinsulinemia accelerates inflammation and increases Aß levels, leading to AD pathogenesis (Fishel et al., 2005; Chatterjee and Mudher, 2018). Schisandra chinensis extract, diapocynin and sodium orthovanadate promote the expression of brain-derived neurotrophic factor gene which promotes neurogenesis, modulates neuronal activity and may downregulate the production of Aß (Buchman et al., 2016). Lychee seed extract, evodiamine and SCR-1693 promotes significant decrease in acetylcholinesterase (AChE) activity. It is known that AChE inhibition is an efficient therapeutic for dementia through the rescue of cholinergic deficit (Eldufani and Blaise, 2019).

Impaired spatial memory is an early clinical sign of AD (Zhu et al., 2017). Compounds seed of litchi chinensis fraction, α-Lipoic acid, sea cucumber cerebrosides, evodiamine, osthole, brain-derived neurotrophic factor, Pureariae Radix, N.incisum extract, Asiatic acid, seed of litchi chinensis fraction, Yonkenafil, sodium orthovanodate, SCR-1693, diapocynin, DL0410, 2-APB, P. harmala (Tang et al., 2018; Wang et al., 2018; Xu et al., 2018; Zhang et al., 2018; Ahmad Rather et al., 2019; Huang et al., 2019; Li et al., 2019; Yao et al., 2019; Zhu et al., 2019; Akhtar et al., 2020; Bi et al., 2020; Ibrahim et al., 2020; Jiang et al., 2020; Sun et al., 2020; Thapak et al., 2020; Zhou et al., 2020; Saleh et al., 2021) showed improvement in spatial memory, which is consistent with the effects of other GSK3 inhibitors such as SB 216763 (Nguyen et al., 2018) or lithium, the first FDA (Food and Drug Administration) approved GSK3 inhibitor (Liu et al., 2020). Compounds yuzu, schisandra chinensis extract, Pureariae Radix and LMDS-1 (Yang et al., 2013; Yan et al., 2017; Huang et al., 2019; Fan et al., 2020) improved short-term memory which is commonly impaired in AD (Della Sala et al., 2012). Loss of long-term memory is also characteristic for AD (Parra et al., 2009) and bee pollen extract showed improvement in long-term memory (Liao et al., 2019).

GSK-3β can phosphorylate tau protein at various sites *in vitro* and in cell culture models and the epitopes are consistent with those found to be hyperphosphorylated in AD brains. Consequently, activation of GSK-3β leads to aggregation of the tau protein (Lei et al., 2011). Activated GSK-3β is also involved in Aβ formation and accumulation in human AD brains because it modulates the cleavage of APP (Lauretti et al., 2020). These mechanisms could be potentially targeted by GSK3 inhibitors. There has consistently been a poor translation of successful therapeutics of pre-clinical animal dementia models to successful interventions in human dementia clinical trials (Franco and Cedazo-Minguez, 2014).Only two compounds included in this review, α-Lipoic acid and curcumin were studied in human clinical trials. Results from one of the trials concluded that α-Lipoic acid can slow cognitive decline (Shinto et al., 2013). Three trials concluded that the administration of curcumin can disaggregate the Aß deposit in the brain (Baum et al., 2008), prevent Aß aggregation (Begum et al., 2008) and improve memory in healthy adults (Small et al., 2018).

Compounds tested in normal ageing animal models did not show cognitive improvement (Jiang et al., 2020; Zhou et al., 2020). Most of the animal models rely on the utilization of genetic mutations associated with familial AD, while the most common form of AD is sporadic AD (LaFerla and Green, 2012). Furthermore, publication bias cannot be ruled out, particularly in animal studies which are unlikely to be registered and may be less likely to be published if results are negative (van der Naald et al., 2020). It was not possible to perform formal statistical analysis due to small sample sizes of studies examining a number of compounds and the conclusion was based on reported *p*-values. The significance of *p*-values is influenced by the sample size, which is often small in animal studies. The search included studies, which investigated the modulation of the GSK3 pathway. Therefore, compounds potentially inhibiting the GSK3 pathway without mentioning the pathway could have been missed. Finally, information on baseline characteristics, outcome assessment, blinding of personnel, housing, allocation concealment, and sequence generation, were often unclear. Future animal trials should consider following the SYRCLE guidelines (Hooijmans et al., 2014).

## 5 Conclusion

The results of this systematic review suggest that the investigated compounds can improve cognitive function in MCI and dementia animal models. Further studies are required to fully elucidate the potential of GSK3 inhibitors in MCI/dementia as well as initiation of clinical trials in humans with compounds inhibiting the GSK3 pathway.
