# Supplementary material for: Targeting Impaired Nutrient Sensing via the Glycogen Synthase Kinase-3 Pathway With Therapeutic Compounds to Prevent or Treat Dementia: A Systematic Review
Source: Front Aging. 2022 Jul 18;3:898853. doi: 10.3389/fragi.2022.898853 (PMC9341294; doi:10.3389/fragi.2022.898853)
Supplement: Supplementary file 1 [file Table1.DOCX]

**Supplementary Table 1.** Results of studies testing the effect of compounds interfering with the GSK3 pathway on cognition.

| Author, year | Cognitive tests | Control (result) | Treatment (result) | Significance |
| --- | --- | --- | --- | --- |
| **Mild cognitive impairment or dementia** | |  |  |  |
| Yang et al., 2013 | **Morris water maze** |  |  |  |
|  | Escape latency (s) 5 day | AD-C 70 (3) | AD-Y 47 (3) | + |
|  | Staying in zone 5 (s) | AD-C 2,45 (0,10) | AD-Y 4,95 (0,10) | + |
|  | Entry latency (min) | AD-C 1,5 (0,10) | AD-Y 2,75 (0,10) | + |
| Madhavadas et al., 2017 | **Open Field Test** |  |  |  |
|  | Distance (cm) | MSG (7m) 1550 (50) | MSG CE 50 mg/kg (7m) 2900 (50) | +++ |
|  | **Barnes maze task** |  |  |  |
|  | Number of errors day 5 (Acquisition) | MSG (7m) 4,8 (0,21) | MSG CE 50 mg/kg (7m) 3,76 (0,22) | + |
|  | Number of errors day 10 (Retention) | MSG (7m) 3,7 (0,48) | MSG CE 50 mg/kg (7m) 3,2 (0,83) | ± |
|  | Latency (s) 5 day (Acquisition) | MSG (7m) 63.25 (5,1) | MSG CE 50 mg/kg (7m) 46.42 (4,46) | ++ |
|  | Latency (s) 10 day (Retention) | MSG (7m) 65,44 (5,03) | MSG CE 50 mg/kg (7m) 47,3 (5,89) | + |
|  | **Open Field Test** |  |  |  |
|  | Distance (cm) | MSG (15m) 1300 (50) | MSG CE 50 mg/kg (15m) 1400 (50) | ± |
|  | **Barnes maze task** |  |  |  |
|  | Number of errors day 5 (Acquisition) | MSG (15m) 5,43 (0,72) | MSG CE 50 mg/kg (15m) 4,4 (0,38) | ± |
|  | Number of errors day 10 (Retention) | MSG (15m) 6,2 (0,77) | MSG CE 50 mg/kg (15m) 6 (0,96) | ± |
|  | Latency (s) 5 day (Acquisition) | MSG (15m) 79.15 (7,15) | MSG CE 50 mg/kg (15m) 71,4 (4,59) | ± |
|  | Latency (s) 10 day (Retention) | MSG (15m) 91,44 (6,91) | MSG CE 50 mg/kg (15m) 87,78 (7,90) | ± |
| Tang et al., 2018 | **Morris water maze** |  |  |  |
|  | Escape latency (s) day 5 | NS+STZ 50 (5) | LSE 0,7 g/kg+STZ 31 (5) | ± |
|  | Time (s) | NS+STZ 0,61 (0,05) | LSE 0,7 g/kg+STZ 1,29 (0,05) | ++ |
|  | Numbers | NS+STZ 245 (0,10) | LSE 0,7 g/kg+STZ 3,9 (0,10) | ++ |
|  | Percentage (%) | NS+STZ 17 (1) | LSE 0,7 g/kg+STZ 28 (1) | + |
|  | **Morris water maze** |  |  |  |
|  | Escape latency (s) day 5 | NS+STZ 50 (5) | LSE 1,4 g/kg+STZ 32 (5) | ± |
|  | Time (s) | NS+STZ 0,61 (0,05) | LSE 1,4 g/kg+STZ 1,59 (0,05) | ++ |
|  | Numbers | NS+STZ 245 (0,10) | LSE 1,4 g/kg+STZ 4 (0,10) | ++ |
|  | Percentage (%) | NS+STZ 17 (1) | LSE 1,4 g/kg+STZ 34 (1) | ++ |
|  | **Morris water maze** |  |  |  |
|  | Escape latency (s) day 5 | NS+STZ 50 (5) | LSE 2,8 g/kg+STZ 12 (5) | ± |
|  | Time (s) | NS+STZ 0,61 (0,05) | LSE 2,8 g/kg+STZ 1,29 (0,05) | ++ |
|  | Numbers | NS+STZ 245 (0,10) | LSE 2,8 g/kg+STZ 4,55 (0,10) | ++ |
|  | Percentage (%) | NS+STZ 17 (1) | LSE 2,8 g/kg+STZ 26 (1) | + |
| Yan et al., 2017 | **Morris water maze** |  |  |  |
|  | Escape latency (s) day 5 | CUMS 37,49 (8,25) | SCE 300 mg/kg 35,14 (8,99) | ± |
|  | Time in target quadrant (s) | CUMS 35 (1) | SCE 300 mg/kg 37 (1) | ± |
|  | **Locomotor activity** |  |  |  |
|  | Locomotor length (cm/5mn) | CUMS 1017 (57) | SCE 300 mg/kg 995 (79) | ± |
|  | Immobility time (s) | CUMS 140 (2) | SCE 300 mg/kg 130 (2) | ± |
|  | **Y-maze test** |  |  |  |
|  | Spontaneous alternation (%) | CUMS 49 (1) | SCE 300 mg/kg 52 (1) | ± |
|  | Number of arm entries | CUMS 25,5 (0,5) | SCE 300 mg/kg 27,5 (0,5) | ± |
|  | **Morris water maze** |  |  |  |
|  | Escape latency (s) day 5 | CUMS 37,49 (8.,5) | SCE 600 mg/kg 19,75 (6,18) | + |
|  | Time in target quadrant (s) | CUMS 35 (1) | SCE 600 mg/kg 47 (1) | + |
|  | **Locomotor activity** |  |  |  |
|  | Locomotor length (cm/5mn) | CUMS 1017 (57) | SCE 600 mg/kg 1205 (84) | ± |
|  | Immobility time (s) | CUMS 140 (2) | SCE 600 mg/kg 100 (2) | + |
|  | **Y-maze test** |  |  |  |
|  | Spontaneous alternation (%) | CUMS 49 (1) | SCE 600 mg/kg 61 (1) | + |
|  | Number of arm entries | CUMS 25,5 (0,5) | SCE 600 mg/kg 30 (0,5) | ± |
|  | **Morris water maze** |  |  |  |
|  | Escape latency (s) day 5 | CUMS 37.49 (8.25) | SCE 1200 mg/kg 21,49 (11,03) | + |
|  | Time in target quadrant (s) | CUMS 35 (1) | SCE 1200 mg/kg 50 (1) | + |
|  | **Locomotor activity** |  |  |  |
|  | Locomotor length (cm/5mn) | CUMS 1017 (57) | SCE 1200 mg/kg 1099 (59) | ± |
|  | Immobility time (s) | CUMS 140 (2) | SCE 1200 mg/kg 105 (2) | + |
|  | **Y-maze test** |  |  |  |
|  | Spontaneous alternation (%) | CUMS 49 (1) | SCE 1200 mg/kg 60 (1) | + |
|  | Number of arm entries | CUMS 25,5 (0,5) | SCE 1200 mg/kg 30 (0,5) | ± |
| Zhang et al., 2018 | **Morris water maze** |  |  |  |
|  | Escape latency (s) day 3 (visible platform) | Vehicle 14 (3) | LA 3 mg/kg 10 (3) | ± |
|  | Escape latency (s) day 7 (hidden platform) | Vehicle 30 (3) | LA 3 mg/kg 7 (3) | + |
|  | Passing Times | Vehicle 0,7 (0,3) | LA 3 mg/kg 2 (0,3) | ++ |
|  | **Open Field Test** |  |  |  |
|  | Total distance (cm) | Vehicle 1900 (50) | LA 3 mg/kg 2000 (50) | ± |
|  | Time spent in the center (s) | Vehicle 2 (0,5) | LA 3 mg/kg 14 (0,5) | ++ |
|  | **NOR** |  |  |  |
|  | Location preference index | Vehicle 53 (2) | LA 3 mg/kg 54 (2) | ± |
|  | Recognition index | Vehicle 51 (3) | LA 3 mg/kg 56 (3) | + |
|  | **Morris water maze** |  |  |  |
|  | Escape latency (s) day 3 (visible platform) | Vehicle 14 (3) | LA 10 mg/kg 6 (3) | ± |
|  | Escape latency (s) day 7 (hidden platform) | Vehicle 30 (3) | LA 10 mg/kg 8 (3) | + |
|  | Passing Times | Vehicle 0,7 (0,3) | LA 10 mg/kg 3,2 (0,3) | ++ |
|  | **Open Field Test** |  |  |  |
|  | Total distance (cm) | Vehicle 1900 (50) | LA 10 mg/kg 2000 (50) | ± |
|  | Time spent in the center (s) | Vehicle 2 (0,5) | LA 10 mg/kg 22,5 (0,5) | ++ |
|  | **NOR** |  |  |  |
|  | Location preference index | Vehicle 53 (2) | LA 10 mg/kg 50 (2) | ± |
|  | Recognition index | Vehicle 51 (3) | LA 10 mg/kg 68 (3) | + |
| Koehler et al., 2019 | **Memory tests** | Pre-treatment | Post-treatment |  |
|  | Control (25 trial) | 0.80 (2) (Probability correct) | 0.85 (2) (Probability correct) | ± |
|  | 1uM TDZD-8 (25 trial) | 0,75 (2) (Probability correct) | 0,85 (2) (Probability correct) | ± |
|  | 1uM TDZD-8 + 100nM Okadaic Acid (25 trial) | 0.81 (2) (Probability correct) | 0.79 (2) (Probability correct) | ± |
|  | 100nM Okadaic Acid (25 trial) | 0,85 (2) (Probability correct) | 0,42 (2) (Probability correct) | + |
| Liao et al., 2019 | **Morris water maze** |  |  |  |
|  | Escape latency (s) day 6 | Sco 1mg/kg 54 (1) | Sco+BP 30 mg/kg 48 (1) | ± |
|  | Time in target quadrant (s) | Sco 1mg/kg 15 (1) | Sco+BP 30 mg/kg 20,5 (1) | ± |
|  | Velocity (cm/s) | Sco 1mg/kg 21 (0,5) | Sco+BP 30 mg/kg 20 (0,5) | ± |
|  | **Y-maze test** |  |  |  |
|  | Spontaneous alternation (%) | Sco 1mg/kg 54 (2) | Sco+BP 30 mg/kg 60,5 (2) | ± |
|  | Total arm entry (no) | Sco 1mg/kg 42,5 (1) | Sco+BP 30 mg/kg 42,5 (1) | ± |
|  | **PAT** |  |  |  |
|  | Latency time (s) Acquisition | Sco 1mg/kg 29 (5) | Sco+BP 30 mg/kg 30 (5) | ± |
|  | Latency time (s) Retention | Sco 1mg/kg 60 (10) | Sco+BP 30 mg/kg 100 (10) | ± |
|  | **Morris water maze** |  |  |  |
|  | Escape latency (s) day 6 | Sco 1mg/kg 54 (1) | Sco+BP 100 mg/kg 37 (1) | + |
|  | Time in target quadrant (s) | Sco 1mg/kg 15 (1) | Sco+BP 100 mg/kg 23 (1) | + |
|  | Velocity (cm/s) | Sco 1mg/kg 21 (0,5) | Sco+BP 100 mg/kg 18,5 (0,5) | ± |
|  | **Y-maze test** |  |  |  |
|  | Spontaneous alternation (%) | Sco 1mg/kg 54 (2) | Sco+BP 100 mg/kg 70 (2) | + |
|  | Total arm entry (no) | Sco 1mg/kg 42,5 (1) | Sco+BP 100 mg/kg 33 (1) | ± |
|  | **PAT** |  |  |  |
|  | Latency time (s) Acquisition | Sco 1mg/kg 29 (5) | Sco+BP 100 mg/kg 40,5 (5) | ± |
|  | Latency time (s) Retention | Sco 1mg/kg 60 (10) | Sco+BP 100 mg/kg 140 (10) | + |
|  | **Morris water maze** |  |  |  |
|  | Escape latency (s) day 6 | Sco 1mg/kg 54 (1) | Sco+BP 300 mg/kg 34 (1) | + |
|  | Time in target quadrant (s) | Sco 1mg/kg 15 (1) | Sco+BP 300 mg/kg 23 (1) | + |
|  | Velocity (cm/s) | Sco 1mg/kg 21 (0,5) | Sco+BP 300 mg/kg 19 (0,5) | ± |
|  | **Y-maze test** |  |  |  |
|  | Spontaneous alternation (%) | Sco 1mg/kg 54 (2) | Sco+BP 300 mg/kg 65 (2) | + |
|  | Total arm entry (no) | Sco 1mg/kg 42,5 (1) | Sco+BP 300 mg/kg 37 (1) | ± |
|  | **PAT** |  |  |  |
|  | Latency time (s) Acquisition | Sco 1mg/kg 29 (5) | Sco+BP 300 mg/kg 30 (5) | ± |
|  | Latency time (s) Retention | Sco 1mg/kg 60 (10) | Sco+BP 300 mg/kg 140 (10) | + |
| Li et al., 2018 | **Morris water maze** |  |  |  |
|  | Escape latency (s) day 5 | Model 34 (1) | Cer-L 14 (1) | ± |
|  | Number of platform crossing | Model 2,2 (0,5) | Cer-L 5 (0,5) | + |
|  | Time in target quadrant (s) | Model 20,5 (0,5) | Cer-L 32 (0,5) | + |
|  | Escape latency (s) day 5 | Model 34 (1) | Cer-H 12 (1) | ± |
|  | Number of platform crossing | Model 2,2 (0,5) | Cer-H 6 (0,5) | + |
|  | Time in target quadrant (s) | Model 20,5 (0,5) | Cer-H 35 (0,5) | + |
|  | Escape latency (s) day 3 | Model 27,5 (2) | Cer 17,5 (2) | + |
| SoukhakLari et al., 2018 | **Passive avoidance learning** |  |  |  |
|  | Explore time (s) | Control 39 (5) | Curcumin 50 mg/kg 50 (5) | ± |
|  | Step Trough Latency (s) | Control 140 (5) | Curcumin 50 mg/kg 260 (5) | ++ |
|  | **Passive avoidance learning** |  |  |  |
|  | Explore time (s) | Control 39 (5) | Curcumin 100 mg/kg 40 (5) | ± |
|  | Step Trough Latency (s) | Control 140 (5) | Curcumin 100 mg/kg 250 (5) | ++ |
| Wang et al., 2018 | **Morris water maze** |  |  |  |
|  | Escape latency (s) day 5 | STZ 20 (1) | STZ/Evo 50 mg/kg 20 (1) | ± |
|  | Number of platform crossing | STZ 2,6 (0,2) | STZ/Evo 50 mg/kg 3 (0,2) | ± |
|  | Time in target quadrant (s) | STZ 12 (0,5) | STZ/Evo 50 mg/kg 15 (0,5) | ± |
|  | Speed (cm/s) | STZ 22 (0,5) | STZ/Evo 50 mg/kg23 (0,5) | ± |
|  | Path length (cm) | STZ 1350 (50) | STZ/Evo 50 mg/kg 1280 (50) | ± |
|  | **NOR** |  |  |  |
|  | Recognition index (test section) | STZ 0,4 (0,02) | STZ/Evo 50 mg/kg 0,45 (0,02) | ± |
|  | Recognition index (training section) | STZ 0,51 (0,02) | STZ/Evo 50 mg/kg 0,505 (0,02) | ± |
|  | **Morris water maze** |  |  |  |
|  | Escape latency (s) day 5 | STZ 20 (1) | STZ/Evo 100 mg/kg 6 (1) | + |
|  | Number of platform crossing | STZ 2,6 (0,2) | STZ/Evo 100 mg/kg 4,1 (0,2) | ++ |
|  | Time in target quadrant (s) | STZ 12 (0,5) | STZ/Evo 100 mg/kg 18 (0,5) | ++ |
|  | Speed (cm/s) | STZ 22 (0,5) | STZ/Evo 100 mg/kg 22 (0,5) | ± |
|  | Path length (cm) | STZ 1350 (50) | STZ/Evo 100 mg/kg 1210 (50) | ± |
|  | **NOR** |  |  |  |
|  | Recognition index (test section) | STZ 0,4 (0,02) | STZ/Evo 100 mg/kg 0,55 (0,02) | ++ |
|  | Recognition index (training section) | STZ 0,51 (0,02) | STZ/Evo 100 mg/kg 0,51 (0,02) | ± |
| Yao et al., 2019 | **Morris water maze** |  |  |  |
|  | Escape latency (s) day 5 | APP/PS1 32 (2) | APP/PS1+OST 25 (2) | ++ |
|  | Number of platform crossing | APP/PS1 1,2 (0,5) | APP/PS1+OST 3,6 (0,5) | + |
|  | Time in target quadrant (s) | APP/PS1 12 (0,5) | APP/PS1+OST 20 (0,5) | ++ |
| Xu et al., 2018 | **Morris water maze** |  |  |  |
|  | Escape latency (s) | OA 22 (1) | OA+BDNF 10 (1) | +++ |
|  | Number of platform crossing | OA 8 (1) | OA+BDNF 20 (1) | +++ |
| Huang et al., 2019 | **Morris water maze** |  |  |  |
|  | Escape latency (s) day 4 | Aβ(25-35)/Saline 44 (1) | Aβ(25-35)/PR 14 (1) | + |
|  | Escape latency (s) | Aβ(25-35)/Saline 43 (1) | Aβ(25-35)/PR 12 (1) | +++ |
|  | Duration in the target quadrant (s) | Aβ(25-35)/Saline 11 (1) | Aβ(25-35)/PR 25 (1) | +++ |
|  | Swimming velocity (cm/s) | Aβ(25-35)/Saline 15 (0,5) | Aβ(25-35)/PR 16 (0,5) | ± |
|  | **Y-maze test** |  |  |  |
|  | Spontaneous alternation (%) | Aβ(25-35)/Saline 50 (3) | Aβ(25-35)/PR 72 (3) | +++ |
|  | **Open Field Test** |  |  |  |
|  | Total moved distance (cm) | Aβ(25-35)/Saline 1260 (50) | Aβ(25-35)/PR 1210 (50) | ± |
|  | Time spent in the central zone (s) | Aβ(25-35)/Saline 16 (5) | Aβ(25-35)/PR 41 (5) | ++ |
|  | Duration in open arm (s) | Aβ(25-35)/Saline 38 (5) | Aβ(25-35)/PR 93 (5) | + |
| Jiang et al., 2020 | **Morris water maze** |  |  |  |
|  | Swimming speed (mm/s) day 6 | APP/PS1 138 (5) | APP/PS1+NRE 140 (5) | ± |
|  | Escape latency (s) day 6 | APP/PS1 46 (2) | APP/PS1+NRE 30 (2) | ++ |
| Rather et al., 2019 | **Elevated Plus Maze** |  |  |  |
|  | Transfer latency time (s) ITL vs RTL | AlCl3+AA (30mg) 44 (2) | AlCl3+AA (30mg) 32 (2) | + |
|  |  | AlCl3+AA (75mg) 40,5 (2) | AlCl3+AA (75mg) 30 (2) | + |
|  |  | AlCl3+AA (150mg) 36 (2) | AlCl3+AA (150mg) 25 (2) | + |
|  | **Radial Arm Maze** |  |  |  |
|  | Mean numbers of errors-reference memory | AlCl3 4,5 (0,2) | AlCl3+AA (75mg) 3,35 (0,2) | + |
|  | Mean numbers of errors-working memory | AlCl3 3 (0,2) | AlCl3+AA (75mg) 2,5 (0,2) | + |
|  | **Open Field Test** |  |  |  |
|  | Number of squares crossed/5minutes Peripheral | AlCl3 9 (0,25) | AlCl3+AA (75mg) 14 (0,25) | ± |
|  | Number of squares crossed/5minutes Central | AlCl3 4 (0,25) | AlCl3+AA (75mg) 4 (0,25) | ± |
|  | Number of activity /5 minutes- Rearing | AlCl3 8 (0,25) | AlCl3+AA (75mg) 12 (0,25) | ± |
|  | Number of activity /5 minutes- Grooming | AlCl3 7 (0,25) | AlCl3+AA (75mg) 9 (0,25) | ± |
| Sun et al., 2020 | **Morris water maze** |  |  |  |
|  | Escape latency (s) day 5 | NS+Aβ(25-35) 53 (3) | SLF 120 mg/kg+Aβ(25-35) 36 (3) | ± |
|  | Numbers(s) | NS+Aβ(25-35) 2,3 (0,3) | SLF 120 mg/kg+Aβ(25-35) 4 (0,3) | + |
|  | Times(s) | NS+Aβ(25-35) 33 (2) | SLF 120 mg/kg+Aβ(25-35) 35,5 (2) | ± |
|  | **Morris water maze** |  |  |  |
|  | Escape latency (s) day 5 | NS+Aβ(25-35) 53 (3) | SLF 240 mg/kg+Aβ(25-35) 27 (3) | ± |
|  | Numbers(s) | NS+Aβ(25-35) 2,3 (0,3) | SLF 240 mg/kg+Aβ(25-35) 4,8 (0,3) | + |
|  | Times(s) | NS+Aβ(25-35) 33 (2) | SLF 240 mg/kg+Aβ(25-35) 40 (2) | ± |
|  | **Morris water maze** |  |  |  |
|  | Escape latency (s) day 5 | NS+Aβ(25-35) 53 (3) | SLF 480 mg/kg+Aβ(25-35) 16 (3) | ± |
|  | Numbers(s) | NS+Aβ(25-35) 2,3 (0,3) | SLF 480 mg/kg+Aβ(25-35) 6,7 (0,3) | ++ |
|  | Times(s) | NS+Aβ(25-35) 33 (2) | SLF 480 mg/kg+Aβ(25-35) 44 (2) | + |
| Zhu et al., 2019 | **Morris water maze** |  |  |  |
|  | Swimming distance (cm) day 5 | icv-STZ+saline 610 (50) | icv-STZ+1 mg/kg yonk 625 (50) | ± |
|  | Escape latency (s) day 5 | icv-STZ+saline 34 (5) | icv-STZ+1 mg/kg yonk 27 (5) | ± |
|  | Swimming speed (cm/s) day 5 | icv-STZ+saline 17,8 (2) | icv-STZ+1 mg/kg yonk 19,5 (2) | ± |
|  | Number of platform crossings | icv-STZ+saline 1,6 (0,5) | icv-STZ+1 mg/kg yonk 3,9 (0,5) | ± |
|  | **Y-maze test** |  |  |  |
|  | Number of arm entries in Y-maze | icv-STZ+saline 21 (1) | icv-STZ+1 mg/kg yonk 21 (1) | ± |
|  | Alternations of arm entries in the Y-maze test (%) | icv-STZ+saline 56 (5) | icv-STZ+1 mg/kg yonk 66 (5) | ± |
|  | **Open Field Test** |  |  |  |
|  | Line crossing | icv-STZ+saline 32 (5) | icv-STZ+1 mg/kg yonk 88 (5) | + |
|  | Rearings (s) | icv-STZ+saline 9 (5) | icv-STZ+1 mg/kg yonk 19 (5) | ± |
|  | **Morris water maze** |  |  |  |
|  | Swimming distance (cm) day 5 | icv-STZ+saline 610 (50) | icv-STZ+3 mg/kg yonk 500 (50) | ± |
|  | Escape latency (s) day 5 | icv-STZ+saline 34 (5) | icv-STZ+3 mg/kg yonk 20 (5) | + |
|  | Swimming speed (cm/s) day 5 | icv-STZ+saline 17,8 (2) | icv-STZ+3 mg/kg yonk 21,1 (2) | ± |
|  | Number of platform crossings | icv-STZ+saline 1,6 (0,5) | icv-STZ+3 mg/kg yonk 4,65 (0,5) | + |
|  | **Y-maze test** |  |  |  |
|  | Number of arm entries in Y-maze | icv-STZ+saline 21 (1) | icv-STZ+3 mg/kg yonk 18 (1) | ± |
|  | Alternations of arm entries in the Y-maze test (%) | icv-STZ+saline 56 (5) | icv-STZ+3 mg/kg yonk 76 (5) | + |
|  | **Open Field Test** |  |  |  |
|  | Line crossing | icv-STZ+saline 32 (5) | icv-STZ+3 mg/kg yonk 92 (5) | + |
|  | Rearings (s) | icv-STZ+saline 9 (5) | icv-STZ+3 mg/kg yonk 18 (5) | ± |
|  | **Morris water maze** |  |  |  |
|  | Swimming distance (cm) day 5 | icv-STZ+saline 610 (50) | icv-STZ+10 mg/kg yonk 310 (50) | + |
|  | Escape latency (s) day 5 | icv-STZ+saline 34 (5) | icv-STZ+10 mg/kg yonk 11 (5) | ++ |
|  | Swimming speed (cm/s) day 5 | icv-STZ+saline 17,8 (2) | icv-STZ+10 mg/kg yonk 19,8 (2) | ± |
|  | Number of platform crossings | icv-STZ+saline 1,6 (0,5) | icv-STZ+10 mg/kg yonk 4,4 (0,5) | + |
|  | **Y-maze test** |  |  |  |
|  | Number of arm entries in Y-maze | icv-STZ+saline 21 (1) | icv-STZ+10 mg/kg yonk 20 (1) | ± |
|  | Alternations of arm entries in the Y-maze test (%) | icv-STZ+saline 56 (5) | icv-STZ+10 mg/kg yonk 80 (5) | ++ |
|  | **Open Field Test** |  |  |  |
|  | Line crossing | icv-STZ+saline 32 (5) | icv-STZ+10 mg/kg yonk 98 (5) | + |
|  | Rearings (s) | icv-STZ+saline 9 (5) | icv-STZ+10 mg/kg yonk 22 (5) | ++ |
| Akhtar et al., 2020 | **Morris water maze** |  |  |  |
|  | Escape latency (s) day 4 | ICV-STZ 3 mg/kg 66 (3) | ICV-STZ+SOV 5 mg/kg 30 (3) | + |
|  | Number of entries in target quadrant | ICV-STZ 3 mg/kg 4 (0,5) | ICV-STZ+SOV 5 mg/kg 6 (0,5) | + |
|  | Time in target quadrant (s) | ICV-STZ 3 mg/kg 13 (1) | ICV-STZ+SOV 5 mg/kg 16 (1) | ± |
|  | **Open Field Test** |  |  |  |
|  | Number of lines crossed | ICV-STZ 3 mg/kg 80 (2) | ICV-STZ+SOV 5 mg/kg 66 (2) | ± |
|  | Number of rearings | ICV-STZ 3 mg/kg 17 (0,5) | ICV-STZ+SOV 5 mg/kg 13 (0,5) | ± |
|  | **NOR** |  |  |  |
|  | Discrimination index | ICV-STZ 3 mg/kg 0,01 (0,05) | ICV-STZ+SOV 5 mg/kg 0,38 (0,05) | + |
|  | **Morris water maze** |  |  |  |
|  | Escape latency (s) day 4 | ICV-STZ 3 mg/kg 66 (3) | ICV-STZ+SOV 10 mg/kg 29 (3) | + |
|  | Number of entries in target quadrant | ICV-STZ 3 mg/kg 4 (0,5) | ICV-STZ+SOV 10 mg/kg 6,3 (0,5) | + |
|  | Time in target quadrant (s) | ICV-STZ 3 mg/kg 13 (1) | ICV-STZ+SOV 10 mg/kg 19 (1) | + |
|  | **Open Field Test** |  |  |  |
|  | Number of lines crossed | ICV-STZ 3 mg/kg 80 (2) | ICV-STZ+SOV 10 mg/kg 70 (2) | ± |
|  | Number of rearings | ICV-STZ 3 mg/kg 17 (0,5) | ICV-STZ+SOV 10 mg/kg 13 (0,5) | ± |
|  | **NOR** |  |  |  |
|  | Discrimination index | ICV-STZ 3 mg/kg 0,01 (0,05) | ICV-STZ+SOV 10 mg/kg 0,5 (0,05) | + |
| Bi et al., 2020 | **Morris water maze** |  |  |  |
|  | Escape latency (s) day 4 | Model 57 (2) | SCR-1693 1 mg/kg 46 (2) | ± |
|  | Time in platform quadrant (%) | Model 35 (2) | SCR-1693 1 mg/kg 40 (2) | ± |
|  | Swimming speed (mm/s) day 4 | Model 29 (2) | SCR-1693 1 mg/kg 28 (2) | ± |
|  | Swimming speed (mm/s) | Model 31 (5) | SCR-1693 1 mg/kg 30 (5) | ± |
|  | **Morris water maze** |  |  |  |
|  | Escape latency (s) day 4 | Model 57 (2) | SCR-1693 2 mg/kg 31 (2) | + |
|  | Time in platform quadrant (%) | Model 35 (2) | SCR-1693 2 mg/kg 40,5 (2) | ± |
|  | Swimming speed (mm/s) day 4 | Model 29 (2) | SCR-1693 2 mg/kg 20 (2) | ± |
|  | Swimming speed (mm/s) | Model 31 (5) | SCR-1693 2 mg/kg 30 (5) | ± |
|  | **Morris water maze** |  |  |  |
|  | Escape latency (s) day 4 | Model 57 (2) | SCR-1693 4 mg/kg 31 (2) | + |
|  | Time in platform quadrant (%) | Model 35 (2) | SCR-1693 4 mg/kg 45 (2) | + |
|  | Swimming speed (mm/s) day 4 | Model 29 (2) | SCR-1693 4 mg/kg 21 (2) | ± |
|  | Swimming speed (mm/s) | Model 31 (5) | SCR-1693 4 mg/kg 30 (5) | ± |
| Ibrahim et al., 2020 | **Morris water maze** |  |  |  |
|  | Escape latency (s) day 5 | D-gal/OVX 38 (2) | Diapo 3 (2) | + |
|  | Time in target quadrant (s) | D-gal/OVX 16 (0,5) | Diapo 33 (0,5) | + |
|  | **NOR** |  |  |  |
|  | Discrimination index | D-gal/OVX -0,45 (0,1) | Diapo 0,6 (0,1) | + |
|  | Recognition index (%) | D-gal/OVX 31 (2) | Diapo 78 (2) | + |
|  | Number of line crossing | D-gal/OVX 90 (3) | Diapo 64 (3) | ± |
|  | Number of rearing | D-gal/OVX 30 (2) | Diapo 25,5 (2) | ± |
| Zhou et al., 2020 | **Morris water maze** |  |  |  |
|  | Latency (s) day 5 | APP/PS1 40 (2,5) | DL0410 3 mg/kg 30 (2,5) | + |
|  | Swimming speed (cm/s) | APP/PS1 8,6 (0,5) | DL0410 3 mg/kg 8,65 (0,5) | ± |
|  | The escape latency (s) | APP/PS1 44 (2) | DL0410 3 mg/kg 30 (2) | ± |
|  | The crossing numbers | APP/PS1 1 (0,50) | DL0410 3 mg/kg 2 (0,5) | ± |
|  | The searching time (s) | APP/PS1 10 (1) | DL0410 3 mg/kg 17 (1) | + |
|  | The searching distance (cm) | APP/PS1 90 (10) | DL0410 3 mg/kg 170 (10) | ++ |
|  | **Morris water maze** |  |  |  |
|  | Latency (s) day 5 | APP/PS1 40 (2,5) | DL0410 10 mg/kg 29 (2,5) | + |
|  | Swimming speed (cm/s) | APP/PS1 8,6 (0,5) | DL0410 10 mg/kg 8,5 (0,5) | ± |
|  | The escape latency (s) | APP/PS1 44 (2) | DL0410 10 mg/kg 17 (2) | + |
|  | The crossing numbers | APP/PS1 1 (0,50) | DL0410 10mg/kg 3 (0,5) | ± |
|  | The searching time (s) | APP/PS1 10 (1) | DL0410 10 mg/kg 16 (1) | ± |
|  | The searching distance (cm) | APP/PS1 90 (10) | DL0410 10 mg/kg 160 (10) | + |
|  | **Morris water maze** |  |  |  |
|  | Latency (s) day 5 | APP/PS1 40 (2,5) | DL0410 30 mg/kg 27,5 (2,5) | + |
|  | Swimming speed (cm/s) | APP/PS1 8,6 (0,5) | DL0410 30 mg/kg 8,7 (0,5) | ± |
|  | The escape latency (s) | APP/PS1 44 (2) | DL0410 30 mg/kg 25 (2) | ++ |
|  | The crossing numbers | APP/PS1 1 (0,50) | DL0410 30 mg/kg 2 (0,5) | ± |
|  | The searching time (s) | APP/PS1 10 (1) | DL0410 30 mg/kg 13 (1) | ± |
|  | The searching distance (cm) | APP/PS1 90 (10) | DL0410 30 mg/kg 130 (10) | ± |
| Fan et al., 2020 | **Open Field Test** |  |  |  |
|  | Total moved distance (cm) | Aβ(25-35) 1300 (50) | LMDS-1 1250 (50) | ± |
|  | Duration in central zone (sec) | Aβ(25-35) 31 (2) | LMDS-1 24 (2) | ± |
|  | **Elevated plus maze** |  |  |  |
|  | Duration in open arms (sec) | Aβ(25-35) 111 (5) | LMDS-1 105 (5) | ± |
|  | **Y-maze test** |  |  |  |
|  | Spontaneous alternative rate (%) | Aβ(25-35) 55 (2) | LMDS-1 68 (2) | + |
| Thapak et al., 2020 | **Y-maze test** |  |  |  |
|  | %Alternation | β-amyloid (Aβ) 55 (3) | Aβ+2-APB3 62 (3) | ± |
|  |  | β-amyloid (Aβ) | Aβ+2-APB10 72 (3) | +++ |
|  | %Time spent in arms (sec) N | β-amyloid (Aβ) 21 (2) | Aβ+2-APB3 25 (2) | ± |
|  |  | β-amyloid (Aβ) 21 (2) | Aβ+2-APB10 46,5 (2) | +++ |
|  | %Time spent in arms (sec) E | β-amyloid (Aβ) 46 (2) | Aβ+2-APB3 40 (2) | ± |
|  |  | β-amyloid (Aβ) 46 (2) | Aβ+2-APB10 28 (2) | ± |
|  | %Time spent in arms (sec) S | β-amyloid (Aβ) 28 (2) | Aβ+2-APB3 31 (2) | ± |
|  |  | β-amyloid (Aβ) 28 (2) | Aβ+2-APB10 23 (2) | ± |
|  | Transfer latency (sec) H | β-amyloid (Aβ) 10 (5) | Aβ+2-APB3 20 (5) | ± |
|  |  | β-amyloid (Aβ) 10 (5) | Aβ+2-APB10 15 (5) | ± |
|  | Transfer latency (sec) L | β-amyloid (Aβ) 10 (5) | Aβ+2-APB3 25 (5) | ± |
|  |  | β-amyloid (Aβ) 10 (5) | Aβ+2-APB10 18 (5) | ± |
|  | Transfer latency (sec) R | β-amyloid (Aβ) 45 (5) | Aβ+2-APB3 80 (5) | ± |
|  |  | β-amyloid (Aβ) 45 (5) | Aβ+2-APB10 230 (5) | + |
|  | **Morris Water Maze** |  |  |  |
|  | Escape latency (sec) day 4 | β-amyloid (Aβ) 35 (3) | Aβ+2-APB3 43 (3) | ± |
|  |  | β-amyloid (Aβ) 35 (3) | Aβ+2-APB10 20 (3) | ± |
|  | Escape latency (sec) day 5 | β-amyloid (Aβ) 40 (3) | Aβ+2-APB3 38 (3) | ± |
|  |  | β-amyloid (Aβ) 40 (3) | Aβ+2-APB10 15 (3) | + |
|  | Distance travelled (M) day 4 | β-amyloid (Aβ) 6,5 (0,5) | Aβ+2-APB3 8 (0,5) | ± |
|  |  | β-amyloid (Aβ) 6,5 (0,5) | Aβ+2-APB10 3 (0,5) | ± |
|  | Distance travelled (M) day 5 | β-amyloid (Aβ) 7 (0,5) | Aβ+2-APB3 5 (0,5) | ± |
|  |  | β-amyloid (Aβ) 7 (0,5) | Aβ+2-APB10 2 (0,5) | ++ |
|  | Mean speed (m/sec) day 4 | β-amyloid (Aβ) 0,18 (0,01) | Aβ+2-APB3 0,17 (0,01) | ± |
|  |  | β-amyloid (Aβ) 0,18 (0,01) | Aβ+2-APB10 0,17 (0,01) | ± |
|  | Mean speed (m/sec) day 5 | β-amyloid (Aβ) 0,18 (0,01) | Aβ+2-APB3 0,16 (0,01) | ± |
|  |  | β-amyloid (Aβ) 0,18 (0,01) | Aβ+2-APB10 0,18 (0,01) | ± |
|  | %Time spent in SW quadrant | β-amyloid (Aβ) 22 (0,5) | Aβ+2-APB3 26 (0,5) | ± |
|  |  | β-amyloid (Aβ) 22 (0,5) | Aβ+2-APB10 42 (0,5) | +++ |
| Saleh et al., 2021 | **Y-maze test** |  |  |  |
|  | %Spontaneous alternation percentage | AlCl3 42 (2) | AlCl3+Ph 71 (2) | + |
|  | Total arm entries | AlCl3 6 (0,5) | AlCl3+Ph 13 (0,5) | + |
|  | Escape latency (sec) day 40 | AlCl3 12 (1) | AlCl3+Ph 8 (1) | ± |
|  | Escape latency (sec) day 41 | AlCl3 14 (1) | AlCl3+Ph 7 (1) | + |
|  | **Morris Water Maze** |  |  |  |
|  | Percent quadrant time (Q) | AlCl3 24 (1) | AlCl3+Ph 36 (1) | + |
| Chou et al., 2021 | **Morris Water Maze** |  |  |  |
|  | Distance in target quadrant (ratio%) | Okadaic acid 25 (3) | Evodiamine 50 32 (3) | + |
|  |  | Okadaic acid 25 (3) | Evodiamine 100 35 (3) | + |
|  | Time in target quadrant (ratio%) | Okadaic acid 24 (3) | Evodiamine 50 31 (3) | + |
|  |  | Okadaic acid 24 (3) | Evodiamine 100 35 (3) | ++ |
|  | Swimming pool (velocity (cm/sec) | Okadaic acid 23 (2) | Evodiamine 50 25 (2) | ± |
|  |  | Okadaic acid 23 (2) | Evodiamine 100 24,5 (2) | ± |
|  | **Passive avoidance learning** |  |  |  |
|  | Transfer latency time (sec) 1^st^ day | Okadaic acid 5 (3) | Evodiamine 50 10 (3) | ± |
|  |  | Okadaic acid 5 (3) | Evodiamine 100 10 (3) | ± |
|  | Transfer latency time (sec) 2^nd^ day | Okadaic acid 15 (3) | Evodiamine 50 110 (3) | ± |
|  |  | Okadaic acid 15 (3) | Evodiamine 100 290 (3) | +++ |
| Lin et al., 2021 | **Morris Water Maze** |  |  |  |
|  | Escape latency (sec) day 4 | APP/PS1 32 (2) | APP/PS1+PPD 32 (2) | ± |
|  |  | APP/PS1 32 (2) | PPP/PS1+OA 27 (2) | ± |
|  | Escape latency (sec) day 5 | APP/PS1 35 (2) | APP/PS1+PPD 24 (2) | + |
|  |  | APP/PS1 35 (2) | PPP/PS1+OA 25 (2) | + |
|  | Time in target quadrant (%) | APP/PS1 12 (2) | APP/PS1+PPD 25 (2) | ++ |
|  |  | APP/PS1 12 (2) | PPP/PS1+OA 29 (2) | ++ |
|  | Platform location crosses | APP/PS1 0,3 (0,1) | APP/PS1+PPD 2,5 (0,1) | + |
|  |  | APP/PS1 0,3 (0,1) | PPP/PS1+OA 2 (0,1) | ± |
|  | Distance (cm) | APP/PS1 850 (20) | APP/PS1+PPD 1020 (20) | ± |
|  |  | APP/PS1 850 (20) | PPP/PS1+OA 1080 (20) | ± |
|  | Velocity (cm/s) | APP/PS1 15 (0,2) | APP/PS1+PPD 17 (0,2) | ± |
|  |  | APP/PS1 15 (0,2) | PPP/PS1+OA 18 (0,2) | ± |
|  | **Y-maze test** |  |  |  |
|  | Time in novel arm (%) | APP/PS1 35 (2) | APP/PS1+PPD 48 (2) | + |
|  |  | APP/PS1 35 (2) | PPP/PS1+OA 47 (2) | + |
|  | Distanced travelled in novel arm (%) | APP/PS1 32 (1) | APP/PS1+PPD 36 (1) | ± |
|  |  | APP/PS1 32 (1) | PPP/PS1+OA 38 (1) | ± |
|  | Entries into novel arm | APP/PS1 3,7 (0,1) | APP/PS1+PPD 3,8 (0,1) | ± |
|  |  | APP/PS1 3,7 (0,1) | PPP/PS1+OA 4,7 (0,1) | ± |
|  | **Open Field Test** |  |  |  |
|  | Distance travelled in center (cm) | APP/PS1 160 (10) | APP/PS1+PPD 220 (10) | ± |
|  |  | APP/PS1 160 (10) | PPP/PS1+OA 240 (10) | ± |
|  | Time in center (%) | APP/PS1 6,8 (0,3) | APP/PS1+PPD 10,8 (0,3) | ± |
|  |  | APP/PS1 6,8 (0,3) | PPP/PS1+OA 11,9 (0,3) | ± |
| Ren et al., 2021 | **Morris Water Maze** |  |  |  |
|  | Escape latency (sec) day 4 | DM 16 (1) | DM/ZBPYR 10 (1) | + |
|  | Escape latency (sec) day 5 | DM 17 (1) | DM/ZBPYR 10 (1) | + |
|  | Escape latency on the 6^th^ day | DM 15,5 (0,5) | DM/ZBPYR 15,25 (0,5) | ± |
|  | Time in seeking platform location (s) | DM 35 (1) | DM/ZBPYR 26 (1) | + |
|  | Time in target quadrant (s) | DM 8 (1) | DM/ZBPYR 10 (1) | + |
|  | Platform location crossings | DM 5,5 (0,5) | DM/ZBPYR 9 (0,5) | + |
|  | Swimming distance (cm) | DM 1400 (50) | DM/ZBPYR 1850 (50) | + |
| Yan et al., 2021 | **Morris Water Maze** |  |  |  |
|  | Swimming speed (cm/s) | icv+Aβ(1-42) 44 (1) | icv+Aβ(1-42)+FOF 100 45 (1) | ± |
|  |  | icv+Aβ(1-42) 44 (1) | icv+Aβ(1-42)+FOF 300 45 (1) | ± |
|  | Escape distance (cm) day 4 | icv+Aβ(1-42) 2170 (50) | icv+Aβ(1-42)+FOF 100 1100 (50) | +++ |
|  |  | icv+Aβ(1-42) 2170 (50) | icv+Aβ(1-42)+FOF 300 1000 (50) | +++ |
|  | Escape distance (cm) day 5 | icv+Aβ(1-42) 2200 (50) | icv+Aβ(1-42)+FOF 100 1200 (50) | +++ |
|  |  | icv+Aβ(1-42) 2200 (50) | icv+Aβ(1-42)+FOF 300 1000 (50) | +++ |
|  | Time in the target quadrant (s) | icv+Aβ(1-42) 26 (1) | icv+Aβ(1-42)+FOF 100 35 (1) | ++ |
|  |  | icv+Aβ(1-42) 26 (1) | icv+Aβ(1-42)+FOF 300 44 (1) | +++ |
|  | Cross platform times | icv+Aβ(1-42) 0,4 (0,1) | icv+Aβ(1-42)+FOF 100 1,9 (0,1) | ++ |
|  |  | icv+Aβ(1-42) 0,4 (0,1) | icv+Aβ(1-42)+FOF 300 2,4 (0,1) | +++ |
|  | **Y-maze test** |  |  |  |
|  | Number of arm entries (no.) | icv+Aβ(1-42) 47,5 (2) | icv+Aβ(1-42)+FOF 100 46 (2) | ± |
|  |  | icv+Aβ(1-42) 47,5 (2) | icv+Aβ(1-42)+FOF 300 48 (2) | ± |
|  | Spontaneous alternation (%) | icv+Aβ(1-42) 0,41 (0,01) | icv+Aβ(1-42)+FOF 100 0,55 (0,01) | + |
|  |  | icv+Aβ(1-42) 0,41 (0,01) | icv+Aβ(1-42)+FOF 300 0,6 (0,01) | ++ |
| Wang et al., 2021 | **Morris Water Maze** |  |  |  |
|  | Latency (s) day 9 | Surgery 40 (0,5) | Surgery+GAS 25 37 (0,5) | + |
|  |  | Surgery 40 (0,5) | Surgery+GAS 50 35 (0,5) | ++ |
|  |  | Surgery 40 (0,5) | Surgery+GAS 100 32 (0,5) | ++ |
|  | Latency (s) day 10 | Surgery 34 (0,5) | Surgery+GAS 25 30 (0,5) | + |
|  |  | Surgery 34 (0,5) | Surgery+GAS 50 28 (0,5) | ++ |
|  |  | Surgery 34 (0,5) | Surgery+GAS 100 26 (0,5) | ++ |
|  | Time spent in the target quadrant (s) | Surgery 11 (1) | Surgery+GAS 25 20 (1) | ++ |
|  |  | Surgery 11 (1) | Surgery+GAS 50 32,5 (1) | ++ |
|  |  | Surgery 11 (1) | Surgery+GAS 100 37,5 (1) | ++ |
|  | Number of platform crossings | Surgery 0,8 (0,1) | Surgery+GAS 25 2,6 (0,1) | ++ |
|  |  | Surgery 0,8 (0,1) | Surgery+GAS 50 2,8 (0,1) | ++ |
|  |  | Surgery 0,8 (0,1) | Surgery+GAS 100 4,4 (0,1) | ++ |
|  | Swim speed (m/s) day 5 | Surgery 1,90 (0,02) | Surgery+GAS 25 1,88 (0,02) | ± |
|  |  | Surgery 1,90 (0,02) | Surgery+GAS 50 1,92 (0,002) | ± |
|  |  | Surgery 1,90 (0,02) | Surgery+GAS 100 1,91 (0,02) | ± |
| **Normal ageing** | |  |  |  |
| Jiang et al., 2020 | **Morris water maze** |  |  |  |
|  | Swimming speed (mm/s) day 6 | WT 141 (5) | APP/PS1 + NRE 140 (5) | ± |
|  | Escape latency (s) day 6 | WT 30 (2) | APP/PS1 + NRE 30 (2) | ± |
| Zhou at al., 2020 | **Morris water maze** |  |  |  |
|  | Latency (s) day 5 | WT 16 (2,5) | DL0410 3 mg/kg 30 (2,5) | +++ |
|  | Swimming speed (cm/s) | WT 8,5 (0,5) | DL0410 3 mg/kg 8,65 (0,5) | ± |
|  | The escape latency (s) | WT 6 (2) | DL0410 3 mg/kg 30 (2) | ± |
|  | The crossing numbers | WT 3 (0,5) | DL0410 3 mg/kg 2 (0,5) | ± |
|  | The searching time (s) | WT 21 (1) | DL0410 3 mg/kg 17 (1) | ± |
|  | The searching distance (cm) | WT 200 (10) | DL0410 3 mg/kg 170 (10) | ± |
|  | **Morris water maze** |  |  |  |
|  | Latency (s) day 5 | WT 16 (2,5) | DL0410 10 mg/kg 29 (2,5) | +++ |
|  | Swimming speed (cm/s) | WT 8,5 (0,5) | DL0410 10 mg/kg 8,5 (0,5) | ± |
|  | The escape latency (s) | WT 6 (2) | DL0410 10 mg/kg 17 (2) | ± |
|  | The crossing numbers | WT 3 (0,5) | DL0410 10 mg/kg 3 (0,5) | ± |
|  | The searching time (s) | WT 21 (1) | DL0410 10 mg/kg 16 (1) | ± |
|  | The searching distance (cm) | WT 200 (10) | DL0410 10 mg/kg 160 (10) | ± |
|  | **Morris water maze** |  |  |  |
|  | Latency (s) day 5 | WT 16 (2,5) | DL0410 30 mg/kg 27,5 (2,5) | +++ |
|  | Swimming speed (cm/s) | WT 8,5 (0,5) | DL0410 30 mg/kg 8,7 (0,5) | ± |
|  | The escape latency (s) | WT 6 (2) | DL0410 30 mg/kg 25 (2) | ± |
|  | The crossing numbers | WT 3 (0,5) | DL0410 30 mg/kg 2 (0,5) | ± |
|  | The searching time (s) | WT 21 (1) | DL0410 30 mg/kg 13 (1) | ± |
|  | The searching distance (cm) | WT 200 (10) | DL0410 30 mg/kg 130 (10) | ± |

+++ favoring intervention, highly significant p<0.001; ++ favoring intervention, significant p<0.01; + favoring intervention, significant p<0.05. ± not significant; 2-APB, 2-Aminoethoxydiphenyl borate; AA, Asiatic acid; AD, Alzheimer disease; Alo, alogliptin benzoate; BDNF, brain-derived neurotrophic factor; BP, bee Pollen Extract; C, control; CE, aqueous extracts of cinnamon; Cer-L the sea cucumber cerebrosides low dose; Cer-H, the sea cucumber cerebrosides high dose; Cer, the sea cucumber cerebrosides; CUMS, chronic unpredictable mild stress; D-gal, galactose; Diapo, diapocynin; DM, diabetes mellitus; Evo, evodiamine; FOF, flavonoids of okra fruit; GAS, gastrodin; ICV, intracerebroventricular; ITL, initial Transfer Latency; LA, α-Lipoic acid; LPS, lipopolysaccharide; LSE, lychee seed extract; MSG, monosodium L-glutamate; NRE, N. incisum extract; NS, normal saline; OA, Oleanolic acid; OST, C15H16O3; OVX, ovariectomy; PR, Puerariae Radix; PPD, 20(S)-protopanaxadiol; RTL, Retention Transfer Latency; SCE, Schisandra chinensis extract; Sco, scopolamine; SLF, litchi chinensis seed fraction incisum extract; SOV, sodium orthovanadate; STZ, streptozotocin; ZBPYR, ZiBuPiYin; WT, wild type; Y, yuzu extract
