# Supplementary material for: Targeting Impaired Nutrient Sensing via the Glycogen Synthase Kinase-3 Pathway With Therapeutic Compounds to Prevent or Treat Dementia: A Systematic Review
Source: Front Aging. 2022 Jul 18;3:898853. doi: 10.3389/fragi.2022.898853 (PMC9341294; doi:10.3389/fragi.2022.898853)
Supplement: Supplementary file 2 [file Table2.docx]

**Supplementary Table 2.** Cognitive / behavioral tests administered in published animal studies.

| **Abbreviation (if applicable)** | **Full name of test** | **Cognitive / behavioral domain(s) measured** |
| --- | --- | --- |
|  | Barnes maze task | Spatial learning and short-term visual memory |
| FST | Forced swimming test | Depression-like behavior |
|  | Elevated plus maze | Anxiety-like behavior |
| MWM | Morris Water Maze | Spatial learning and short-term visual memory |
| NOR | Novel Object Recognition task | Recognition memory |
| OFT | Open Field Test | Locomotor activity, exploration habits, anxiety-like behavior |
| PAT | Passive avoidance test | Avoidance learning and associative memory (short term or long term) |
|  | Radial Arm Maze | Spatial learning and short-term visual memory |
| SPT | Sucrose preference test | Depression-like behavior |
|  | Y maze task | Spatial learning and short-term visual memory |
